# Standardized Flanged Intrascleral Intraocular Lens Fixation with the Double-Needle Technique for Cataract Luxation in the Vitreous Chamber during Phacoemulsification

**DOI:** 10.1155/2021/9998482

**Published:** 2021-04-28

**Authors:** Gianluca Besozzi, Chiara Posarelli, Maria Carmela Costa, Alessio Montericcio, Giuseppe Nitti, Ermete Giancipoli, Milena L'Abbate, Francesco Pignatelli, Barbara Parolini, Michele Figus

**Affiliations:** ^1^Department of Ophthalmology, Vito Fazzi Hospital, Lecce, Italy; ^2^Ophthalmology, Department of Surgical Medical Molecular Pathology and of Critical Area, University Hospital of Pisa, Pisa, Italy; ^3^Department of Biomedical Sciences, Humanitas University, Milan, Italy; ^4^Department of Ophthalmology, San Giuseppe Moscati Hospital, Taranto, Italy; ^5^Ophthalmology, Eye Care Clinic, Brescia, Italy

## Abstract

**Purpose:**

To assess the visual and refractive outcome of immediate intraoperative vitrectomy and intrascleral intraocular lens implantation using a “standardized” sutureless Yamane technique during cataract luxation in the vitreous chamber as a complication of phacoemulsification.

**Design:**

A prospective, interventional, consecutive case series.

**Materials and Methods:**

Twelve patients underwent vitrectomy and intrascleral intraocular lens fixation using a standardized Yamane technique as the primary procedure during complicated phacoemulsification. Patients were evaluated preoperatively and 6 months postoperatively for best-corrected distance visual acuity, correspondence to the preoperative refractive target in the spherical equivalent, endothelial cell count, and complications.

**Results:**

Mean preoperative best-corrected visual acuity was 1.16 ± 0.3 logarithm of the minimum angle of resolution (logMAR), the endothelial cell count was 1910.5 ± 297.64, and target refraction at baseline was −0.197 ± 0.087. Postoperatively, best-corrected visual acuity was significantly improved; the mean value was 0.05 logMAR ± 0.06. Mean baseline target refraction in the spherical equivalent was −0.20 ± −0.09 (range: −0.08 to −0.37), and mean final refraction was −0.44 ± −0.14 (range: −0.25 to −0.75) with no significant difference (*p*=0.87). No complication was registered intra- and postoperatively.

**Conclusion:**

Standardization of the Yamane technique seemed a valuable option for patients who had complicated phacoemulsification to achieve a predictable refractive outcome. *Synopsis*. The predictable refractive outcome could be achieved with the immediate standardized Yamane technique in patients with intraoperative cataract luxation in the vitreous chamber during phacoemulsification.

## 1. Introduction

The intraocular lens (IOL) scleral fixation using the Yamane technique was introduced in 2014. The technique was sutureless, with an intrascleral fixation and with a good wound closure [1]. There is currently no complete consensus on how to surgically treat aphakia without capsular support. Scleral fixation of a posterior chamber intraocular lens with sutures and retropupillary and anterior chamber fixation of an iris-claw IOL are the alternative surgical techniques for the treatment of aphakia [2–5].

Yamane technique, despite many advantages, is still challenging for many surgeons. First of all, threading the trailing haptic into the needle represented a crucial point for a good scleral fixation. Furthermore, the material of the lens's haptic is an essential factor to be considered to prevent kinking or breakage and therefore failure of the technique [6, 7].

Finally, since it is difficult to insert the haptics into the scleral tunnel using this approach, scleral fixation of the IOL haptics is a significant issue [6–11]. The technique is often poorly standardized in terms of tunnel construction, position of the opposite tunnel, and length of the portion of the haptic that requires cauterization.

Standardization of this procedure may be crucial in achieving the desired refractive target since we must note that these patients were originally cataract patients. In this study, we propose a standardization of the original technique with the aim of improving the final visual outcome of our patients and to overcome all potential surgery challenges.

## 2. Materials and Methods

This is a prospective, interventional, nonrandomized case series. The study was conducted according to the principles defined in the Declaration of Helsinki and amendments. The study was approved by the institutional review board. All patients gave written informed consent after explanation of the nature and possible consequences of the surgery. We evaluated 12 eyes of 12 consecutive patients, who experienced cataract luxation in the vitreous chamber during phacoemulsification with a temporal 2.4 mm clear cornea incision performed by several surgeons. All the patients immediately underwent 23-gauge (g) three-port pars plana vitrectomy (PPV), removal of cataract remnants, and intrascleral fixation using a “standardized” Yamane technique performed by the same expert surgeon (GB).

We evaluated the preoperative data present in the routine cataract surgery charts, such as best-corrected visual acuity in logMAR (BCVA), slit-lamp biomicroscopy, Goldmann tonometry, endothelial cell count (ECC), biometry (IOL Master 700, Carl Zeiss Meditec, Germany), fundus examination after pupil dilation, and macular status assessed by optical coherence tomography (OCT) (Stratus OCT, Carl Zeiss Meditec). All patients completed 6-months of follow-up. We recorded intraoperative complications, and we postoperatively analyzed the correspondence between target refraction and final refraction in the spherical equivalent (SE), BCVA, ECC, and the presence of complications such as iris capture, early postoperative hypotony, anterior chamber or vitreous hemorrhage, CME, or IOL dislocations/decentration.

### 2.1. Surgical Technique

**Figure 1:**
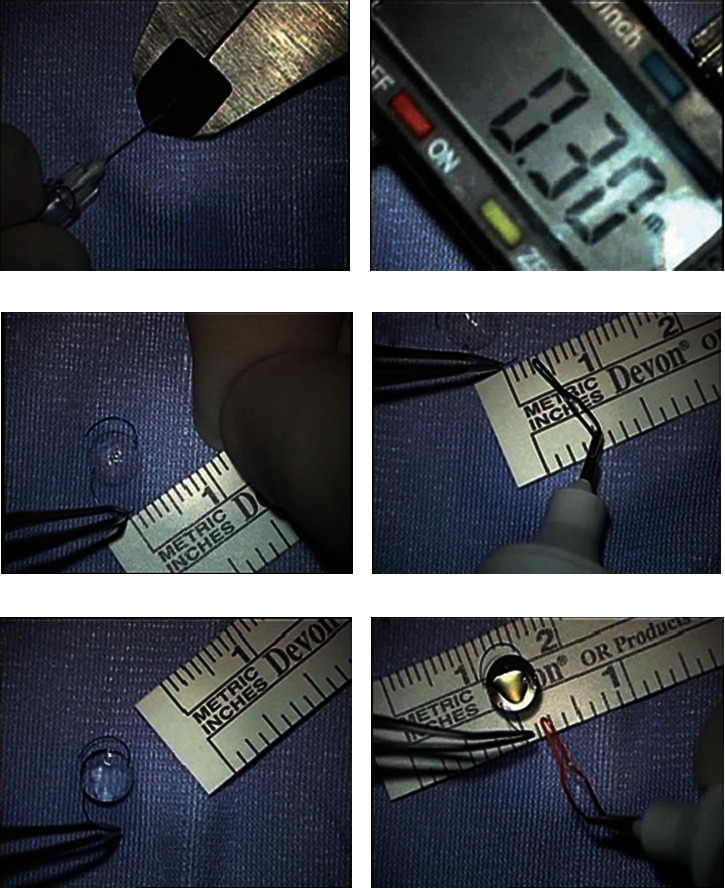
A professional ruler was used to measure the external dimension of the 30 g UTW needle and blocked in that position (a, b) in order to measure the internal dimension of the scleral tunnel. Forceps was put at 1 mm and 2 mm (c, d) from the tip, and the tip was heated from distance until the plug reached the forceps (e, f).

Immediately after cataract luxation in the vitreous chamber, a single experienced surgeon (GB) performed a standard 23 g three-port PPV (Constellation, Alcon, USA) with cataract fragment removal with core vitrectomy, posterior hyaloidectomy, peripheral vitreous shaving without indentation, anterior hyaloidectomy, removal of residual capsular and zonular remnants and scleral fixation IOL implant with “standardized” Yamane's technique, and peripheral retina check with indentation. A superior 2.75 mm clear cornea incision was performed at 11 o'clock, and a corneal paracentesis with a 20 g blade was made opposite to the primary temporal corneal incision. The anterior chamber was filled with dispersive viscoelastic (IAL F, Bausch & Lomb) in order to provide the best protection of the endothelium while also increasing the anterior chamber space. Furthermore, the intraocular pressure (IOP) control of the vitrectomy machine was set to 10 mmHg to prevent hypotony during surgical maneuvers. A dedicated surgical tool called Yamane double-needle stabilizer (Geuder AG, Germany) was used to guide the needle to create the transconjunctival sclerotomies. The stabilizer is designed with several “teeth” underneath the crown for 270 degrees in order to grab the limbal tissue; the remaining 90 degrees with no “teeth” must be applied in the projection of the 2.75 mm superior incision in order to exert no pressure on the main incision and not to lose the stability of the anterior chamber. Setting the IOP at 10 mmHg leads to no iris prolapse throughout the corneal wounds during the scleral tunnel construction. To create the “L-shaped” scleral tunnel, a 30 g ultrathin-wall (UTW) needle (TSK, Japan) was used, and it was bent 7 mm far from the tip in order to be locked into the Yamane stabilizer after creating a 2 mm intrascleral tunnel starting 2 mm far from the limbus. A second scleral tunnel was created in the same fashion. The Yamane stabilizer assures the insertion of the needle at 180 degrees one opposite to the other. At this point, the tip of both needles is free-floating in the vitreous chamber, and the peculiar shape of the tunnel does not allow the tips to get in contact with the retinal surface. A preloaded foldable 3-piece IOL (Kowa PU6AS, Japan) was slowly injected into the viscoelastic-filled anterior chamber through the superior tunnel. The power selected for the IOL implanted was the one measured by automated biometry planned to achieve the negative refraction nearest to 0 if the IOL was placed into the bag. The distal haptic of the IOL was gently placed on the anterior surface of the iris, while the proximal haptic was left outside the tunnel in order to avoid the IOL to fall in the vitreous chamber. Then, using a 23 g vitreoretinal forceps, the distal haptic is placed inside the lumen of the needle, and the same procedure was made for the proximal haptic; the haptics were then externalized through the scleral tunnels as it was previously described. 2 mm was measured using a common ruler from the end of the haptic. We heated 2 mm of the haptic because we previously measured with a professional ruler (Borletti, Italy) the external dimension of the 30 g UTW needle in order to measure the internal dimension of the scleral tunnel. We stuck the ruler at that value. We applied cauterization to the haptics of the IOL, putting the forceps at different distances from the tip in order to investigate the optimal dimension of the plug to be stuck into the scleral tunnel. We put the forceps at 1 mm and 2 mm from the tip and heated the tip from distance. So, we created 2 different dimensions of the plug. The plug created at 1 mm slipped into the ruler, while the plug created at 2 mm does not. It means, to us, that this was the optimal amount of the tip to be heated in order to create a plug that could be inserted into the scleral tunnel by applying a little of stretch at the beginning of the tunnel, but since it is a little bigger than the tunnel, once inside, it will remain stable without slipping (Figure 1). Forceps held the haptic at 2 mm from the tip, and the plug was created by heating the end of the haptic, without touching the haptic. The plugs were gently inserted into the scleral tunnel, and the overlying conjunctiva was mobilized. The trocars were then removed, and the sclerotomies were sealed using wet-field diathermy. The corneal wounds were at the end hydrosutured.

### 2.2. Statistical Analysis

Data were organized in a Microsoft Excel XP table to perform statistical analysis. The Kolmogorov–Smirnov test was used to assess normality of the data. Changes at follow-up were calculated as the difference between follow-up and baseline measurements and were analyzed by Student's *T*-test for paired data assessing differences in mean values; when parametric analysis was not indicated, Wilcoxon signed-rank test was applied to assess the significance of differences between examinations. A *p* value <0.05 was considered statistically significant. Data were presented as mean + standard deviation. Analyses were performed using SPSS (version 21, IBM Corp).

## 3. Results

Twelve consecutive patients were prospectively enrolled and evaluated. Mean age of our patients was 76.42 ± 5.91; 8 were male and 4 were female.

**Figure 2:**
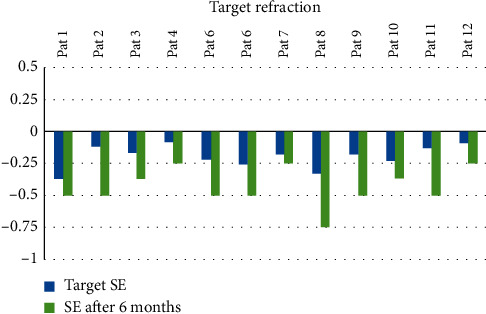
The spherical equivalent (SE) before and after IOL scleral fixation is shown. The difference between the mean preoperative target SE and the mean postoperative SE was not significant (*p*=0.87).

Mean baseline target refraction (SE) was −0.20 ± −0.09 (range: −0.08 to −0.37), and mean final refraction was −0.44 ± −0.14 (range: −0.25 to −0.75).  Figure 2 shows the postoperative spherical equivalent relative to the intended target; we found no significant difference between mean preoperative target SE and mean postoperative SE (*p*=0.87, not significant).

Best-corrected visual acuity increased significantly from 1.16 ± 0.3 logMAR to 0.05 ± 0.06 logMAR (*p* < 0.05).

Mean endothelial cell count at baseline was 1910.5 ± 297.64, and 6 months after IOL scleral fixation, it was 1508.8 ± 294.14 (*p* < 0.05).

All 12 patients had no intraoperative complications such as hemorrhages, haptic damage, or IOL luxation in the vitreous chamber. No hypotony was observed in early postoperative days. At six months, we observed no iris capture, early postoperative hypotony, anterior chamber or vitreous hemorrhage, cystoid macular edema (CME), or IOL dislocations/decentration.

## 4. Discussion

The aim of this prospective nonrandomized series was to demonstrate that an improved standardization of the Yamane technique could lead to a more predictable visual outcome in patients who needed this procedure to immediately repair an intraoperative complication during standard phacoemulsification.

The main and the new advantage related to this further standardization of the technique are related with the final position of the IOL, which did not interfere with the final refractive outcome.

Traditionally, IOL power calculations are based on IOL localization, but to date, there is no consensus on the target spherical equivalent to use when implanting a scleral-fixated intraocular lens [12, 13]. Yamane, in 2017, used four different models of IOLs, and the mean refractive difference from the predicted value differed significantly among the models. The difference from the predicted value was neither related with the tilting of the lens nor with iris capture [6]. However, transscleral fixation of the lens determined a mean myopic shift of −1 diopter because the lens is fixed forward in the eye [12–14].

In our series, we observed no statistically significant difference between the predicted SE for the bag fixation and the final target refraction after 6 months (*p* > 0.05). We speculate that the correspondence with the preoperative target refractive error and the final refractive error could be subsequent to an improvement of the standardization of the technique. The Yamane stabilizer provides a perfect 180° opposition between the scleral tunnel. Bending the 30 g UTW needle at 7 mm from the tip ensures that the surgeon will achieve a 2 mm intrascleral tunnel because the device itself is designed in order to stop the needle when a 2 mm tunnel is created. Another variable we standardized is the amount of the haptics that need to be heated. There are no guidelines about the amount of the material of the haptics that must be cauterized in order to achieve stability of the IOL. We highlight that heating 2 mm of the IOL haptics could be enough to prevent them from slipping out of the 2 mm scleral tunnel and that standardizing the amount of the heated haptic may lead to a predictable refractive outcome. It is important to underline that our findings could only be applied to the selected IOL, as different haptics, even if made of the same material, showed different behaviors if heated [9].

In terms of complications, a reduction in the mean central endothelial cell count after 6 months has been observed from 1910.5 ± 297.64 to 1508.8 ± 294.14. A mean reduction of 22% of endothelial cell count has been already described by other authors after six months [14], but compared with that obtained by Yamane is greater [6]. Nevertheless, in our series, the baseline endothelial cell count was lower, and this could explain the higher loss of endothelial cells after six months. Indeed, the reduction of endothelial cell count did not seem to negatively affect the visual outcome of our patients at all. Furthermore, we did not observe any cystoid macular edema at six months from surgery in contrast with other techniques, such as anterior or posterior iris-claw IOL fixation where CME was observed to be the most frequent complications at both one month and one year of follow-up [15].

Moreover, in our series, we did not observe iris capture, early postoperative hypotony, anterior chamber or vitreous hemorrhage, or IOL dislocations/decentration.

The main limitations of this prospective nonrandomized study are related with the small sample size.

## 5. Conclusion

In conclusion, we observed that the standardization of the Yamane technique using the Yamane stabilizer allows to create sclerotomies at exactly 180°, one opposite to the other, with a predictable geometry of the scleral tunnel. Furthermore, standardizing the type of the IOL and the amount of the haptic that should be heated may lead to a predictable and congruent refractive result in patients that need a sutureless scleral-fixated IOL as an immediate procedure when they experience a cataract luxation in the vitreous chamber during phacoemulsification.

## Data Availability

The supporting data are not available since the data collection was only available on the paper and not electronically.
